# Supplementary material for: Telomeric amplicons of SUL1 and Y’ in yeast are generated by microhomology-mediated break induced replication occurring in cis
Source: PLoS Genet. 2026 Aug 20;22(8):e1012214. doi: 10.1371/journal.pgen.1012214 (PMC13515017; doi:10.1371/journal.pgen.1012214)
Supplement: Supplementary file 1 [file pgen.1012214.s001.pdf]

S1 Fig

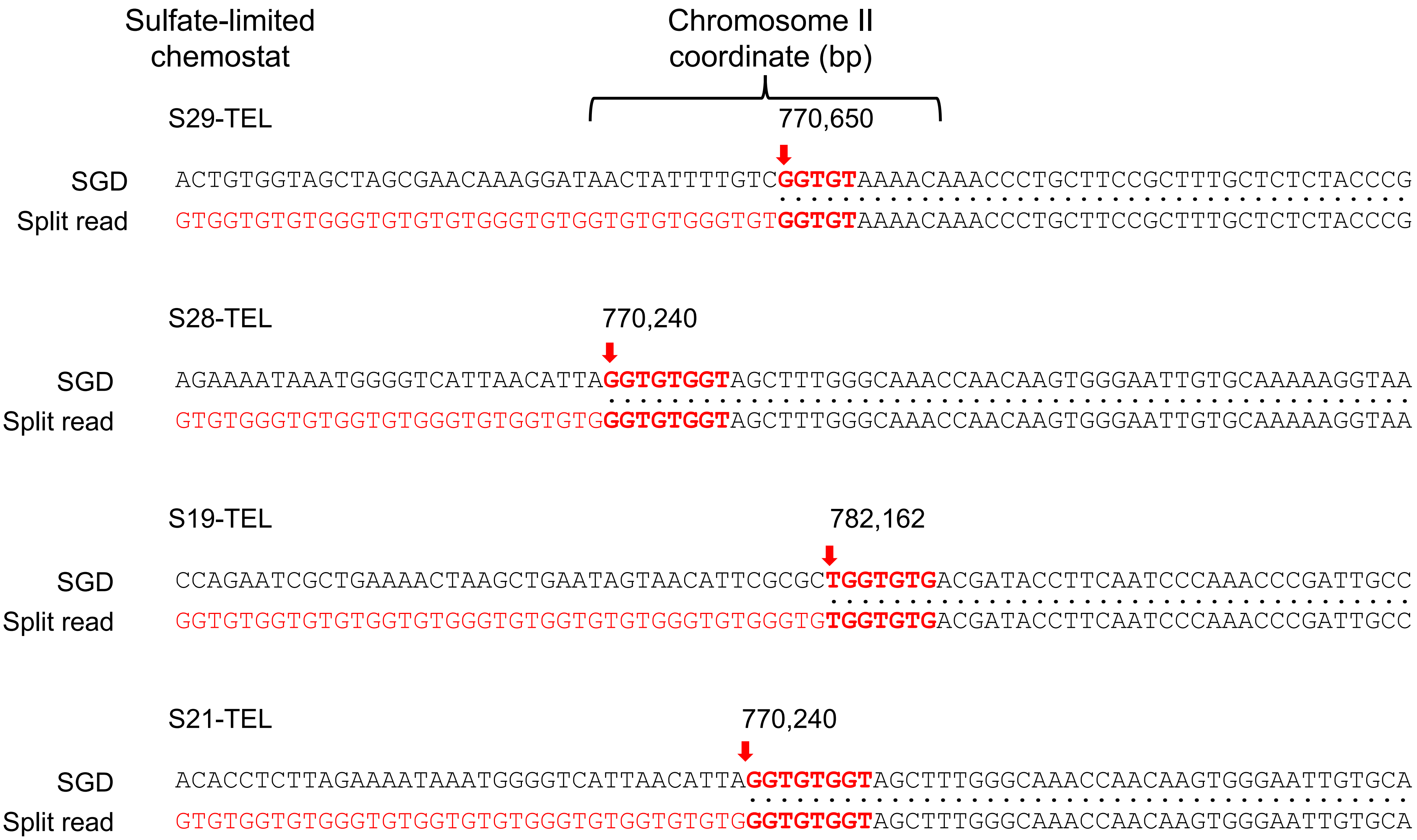
Sulfate-limited chemostat
Chromosome II coordinate (bp)
S29-TEL
770,650
SGD
ACTGTGGTAGCTAGCGAACAAAGGATAACTATTTTGTCGGTGTAAAACAAACCCTGCTTCCGCTTTGCTCTCTACCCG
Split read
GTGGTGTGTGGGTGTGTGTGGGTGTGGTGTGTGGGTGTGGTGTAAAACAAACCCTGCTTCCGCTTTGCTCTCTACCCG
S28-TEL
770,240
SGD
AGAAAATAAATGGGGTCATTAACATTAGGTGTGGTAGCTTTGGGCAAACCAACAAGTGGGAATTGTGCAAAAAGGTAA
Split read
GTGTGGGTGTGGTGTGGGTGTGGTGTGGGTGTGGTAGCTTTGGGCAAACCAACAAGTGGGAATTGTGCAAAAAGGTAA
S19-TEL
782,162
SGD
CCAGAATCGCTGAAAACTAAGCTGAATAGTAACATTCGCGCTGGTGTGACGATACCTTCAATCCCAAACCCGATTGCC
Split read
GGTGTGGTGTGTGGTGTGGGTGTGGTGTGTGGGTGTGGGTGTGGTGTGACGATACCTTCAATCCCAAACCCGATTGCC
S21-TEL
770,240
SGD
ACACCTCTTAGAAAATAAATGGGGTCATTAACATTAGGTGTGGTAGCTTTGGGCAAACCAACAAGTGGGAATTGTGCA
Split read
GTGTGGTGTGGGTGTGGTGTGTGGGTGTGGTGTGTGGGTGTGGTAGCTTTGGGCAAACCAACAAGTGGGAATTGTGCA
