# Supplementary material for: Telomeric amplicons of SUL1 and Y’ in yeast are generated by microhomology-mediated break induced replication occurring in cis
Source: PLoS Genet. 2026 Aug 20;22(8):e1012214. doi: 10.1371/journal.pgen.1012214 (PMC13515017; doi:10.1371/journal.pgen.1012214)
Supplement: Supplementary file 2 [file pgen.1012214.s002.pdf]

S2 Fig

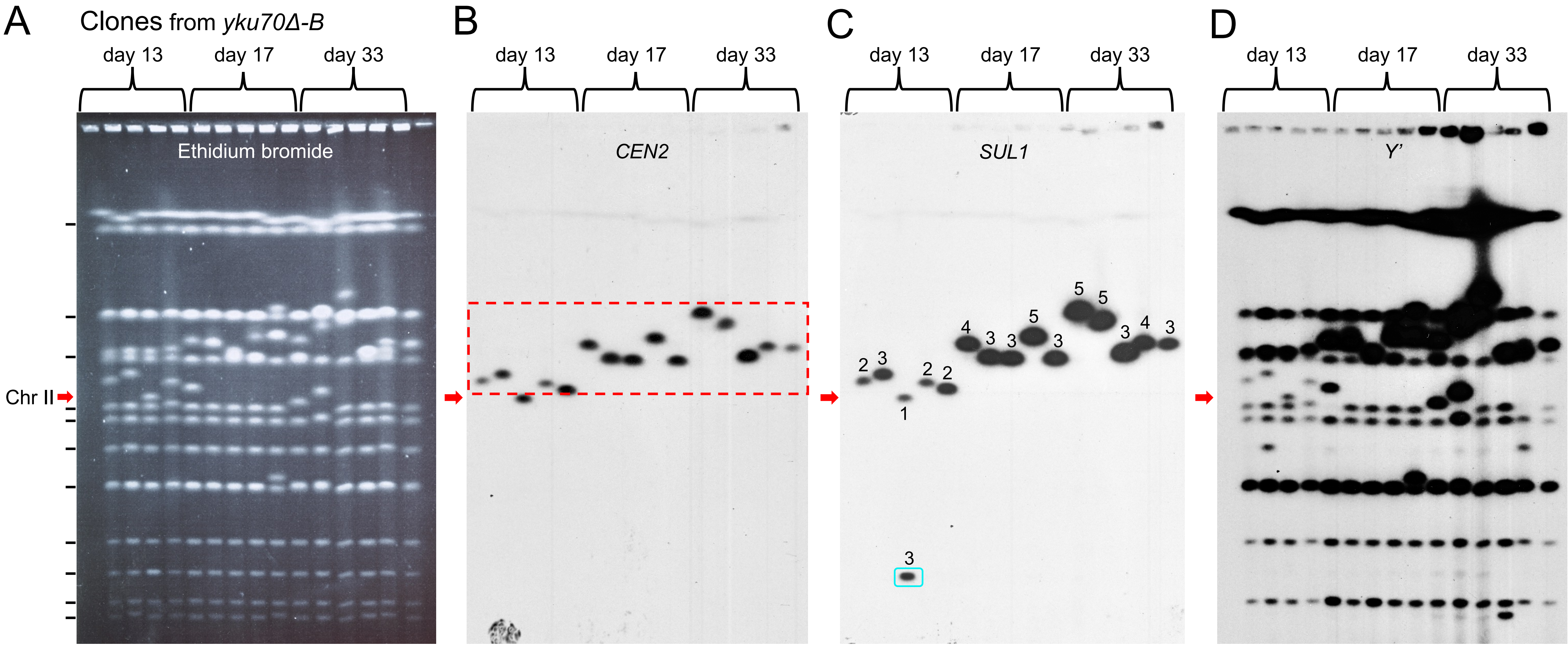

A
Clones from *yku70Δ-B*
day 13
day 17
day 33
Ethidium bromide
Chr II
B
CEN2
C
SUL1
2
3
1
2
2
4
3
3
5
3
5
5
3
4
3
3
D
Y'
