# Supplementary material for: Telomeric amplicons of SUL1 and Y’ in yeast are generated by microhomology-mediated break induced replication occurring in cis
Source: PLoS Genet. 2026 Aug 20;22(8):e1012214. doi: 10.1371/journal.pgen.1012214 (PMC13515017; doi:10.1371/journal.pgen.1012214)
Supplement: Supplementary file 3 [file pgen.1012214.s003.pdf]

S3 Fig

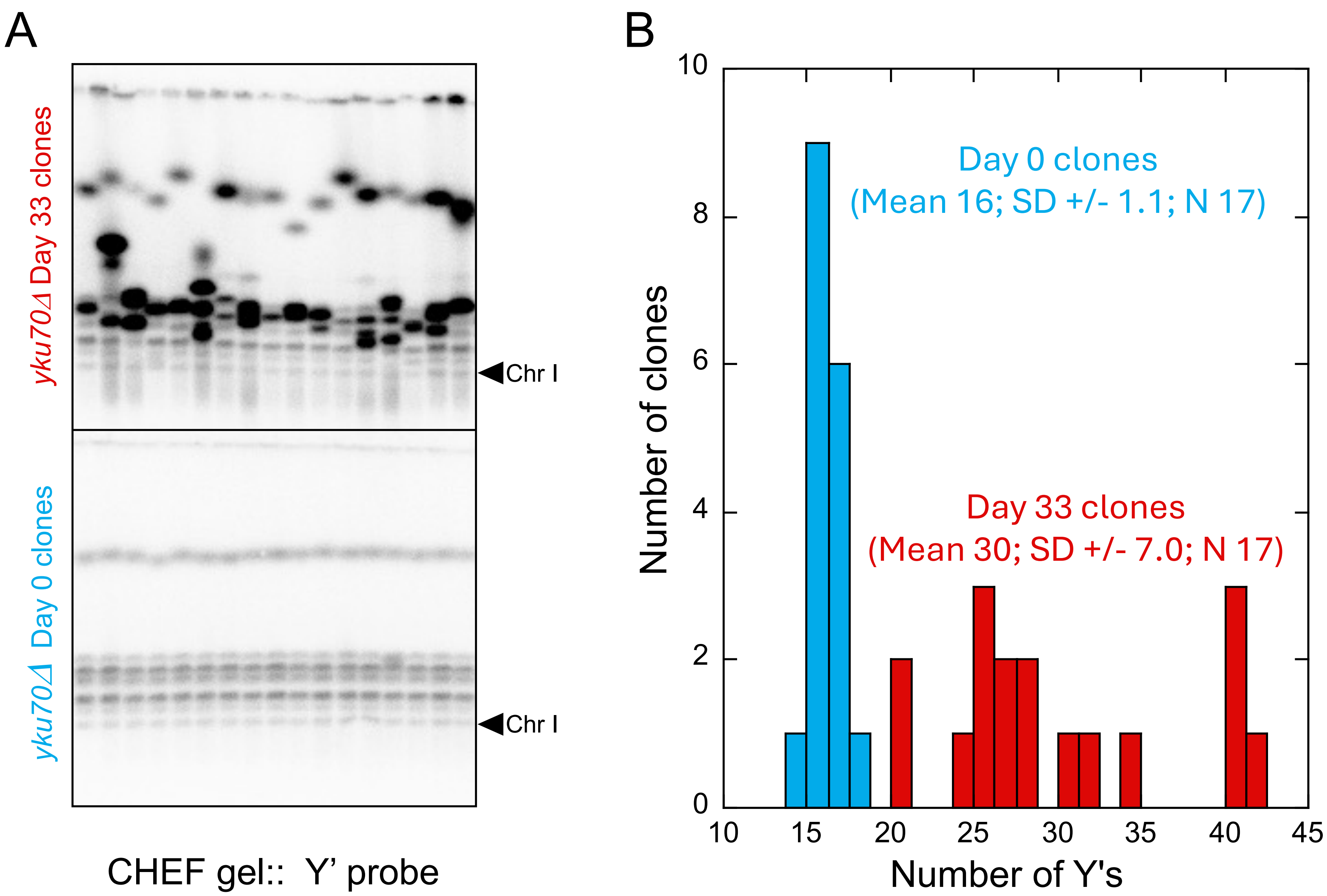

A
yku70Δ Day 33 clones
yku70Δ Day 0 clones
Chr I
Chr I
CHEF gel:: Y' probe
B
Day 0 clones
(Mean 16; SD +/- 1.1; N 17)
Day 33 clones
(Mean 30; SD +/- 7.0; N 17)
Number of clones
Number of Y's
0
2
4
6
8
10
10
15
20
25
30
35
40
45
