# Supplementary material for: Telomeric amplicons of SUL1 and Y’ in yeast are generated by microhomology-mediated break induced replication occurring in cis
Source: PLoS Genet. 2026 Aug 20;22(8):e1012214. doi: 10.1371/journal.pgen.1012214 (PMC13515017; doi:10.1371/journal.pgen.1012214)

S4 Fig

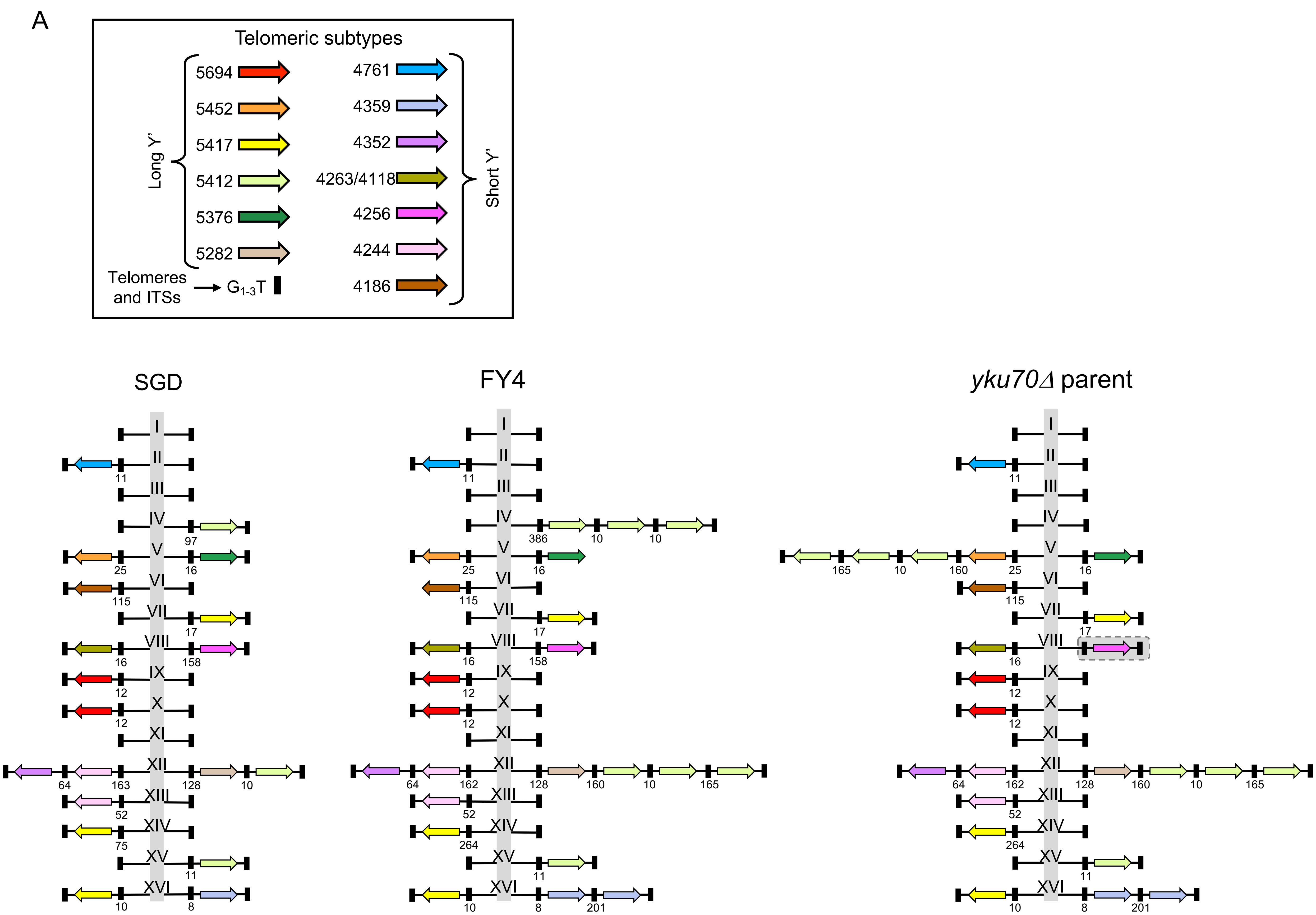
A
Telomeric subtypes
Long Y'
5694
5452
5417
5412
5376
5282
Short Y'
4761
4359
4352
4263/4118
4256
4244
4186
Telomeres and ITSs
G1-3T
SGD
FY4
yku70Δ parent
I
II
III
IV
V
VI
VII
VIII
IX
X
XI
XII
XIII
XIV
XV
XVI

S4 Fig

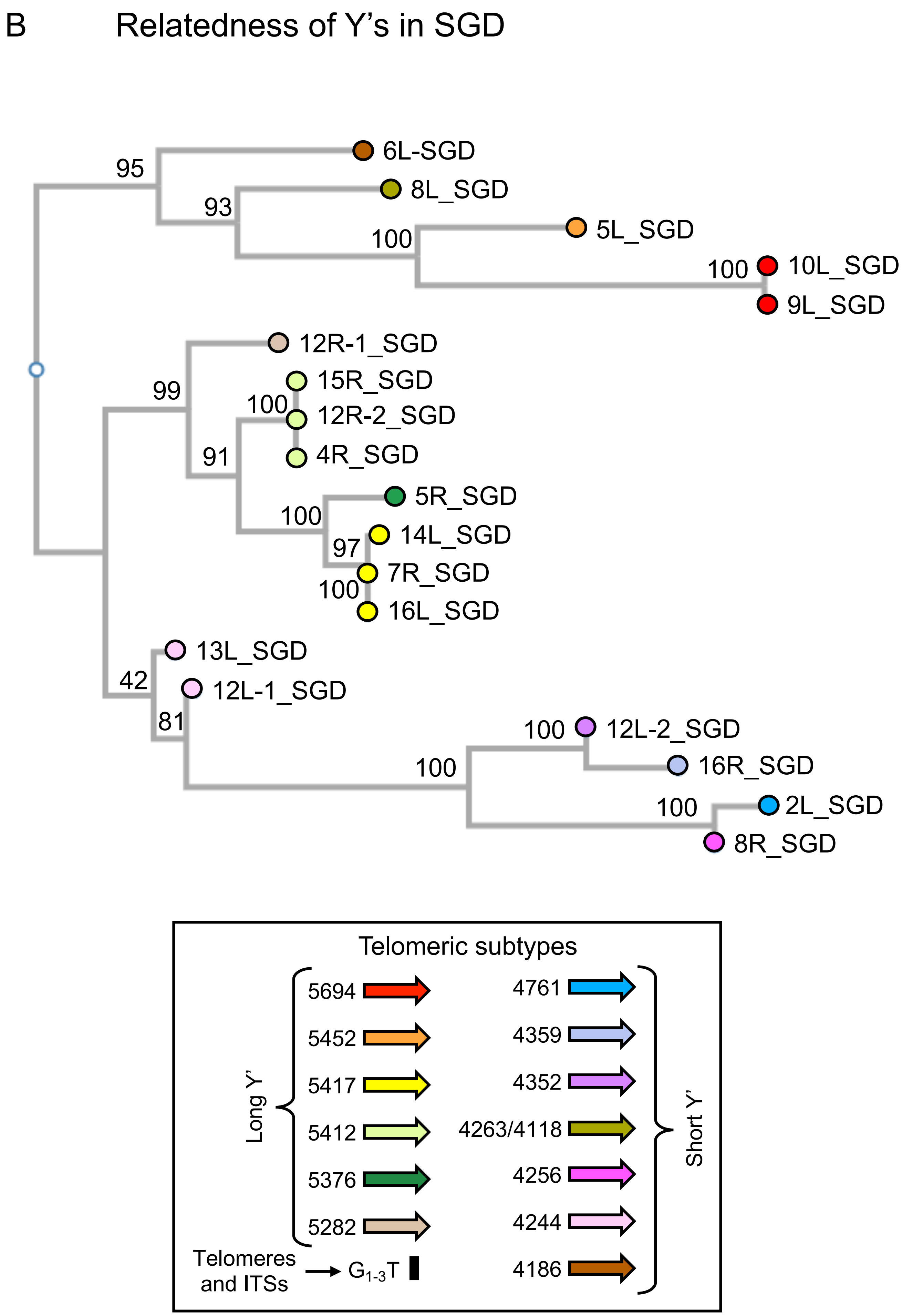
B
Relatedness of Y's in SGD
95
93
100
100
6L-SGD
8L_SGD
5L_SGD
10L_SGD
9L_SGD
99
100
91
100
97
100
12R-1_SGD
15R_SGD
12R-2_SGD
4R_SGD
5R_SGD
14L_SGD
7R_SGD
16L_SGD
42
81
100
100
100
13L_SGD
12L-1_SGD
12L-2_SGD
16R_SGD
2L_SGD
8R_SGD
Telomeric subtypes
Long Y'
5694
5452
5417
5412
5376
5282
Short Y'
4761
4359
4352
4263/4118
4256
4244
4186
Telomeres and ITSs
G1-3T

S4 Fig

C Y’s of FY4 compared to SGD

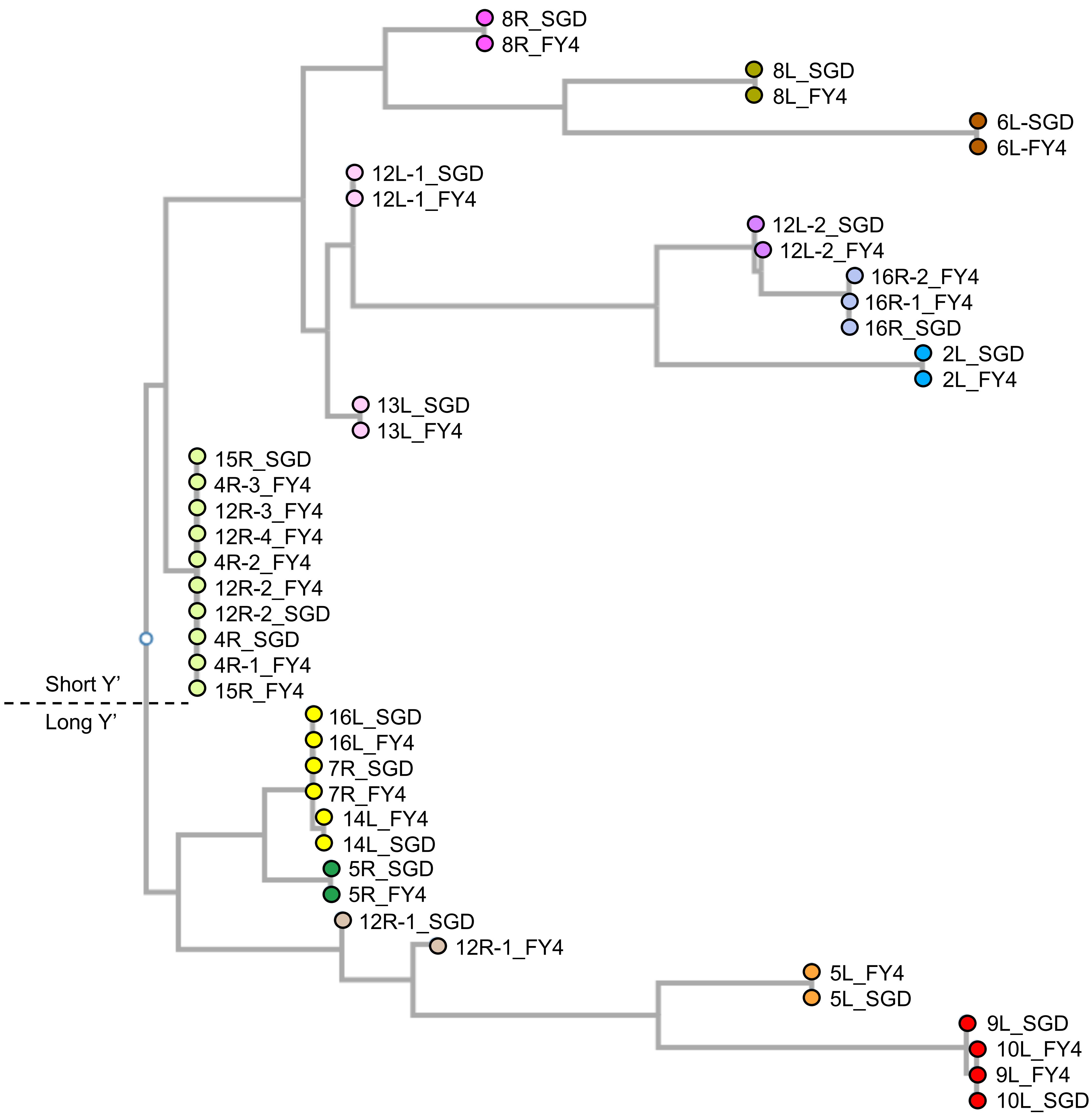

S4 Fig

D   Y's of *yku70Δ* parent strain compared to SGD

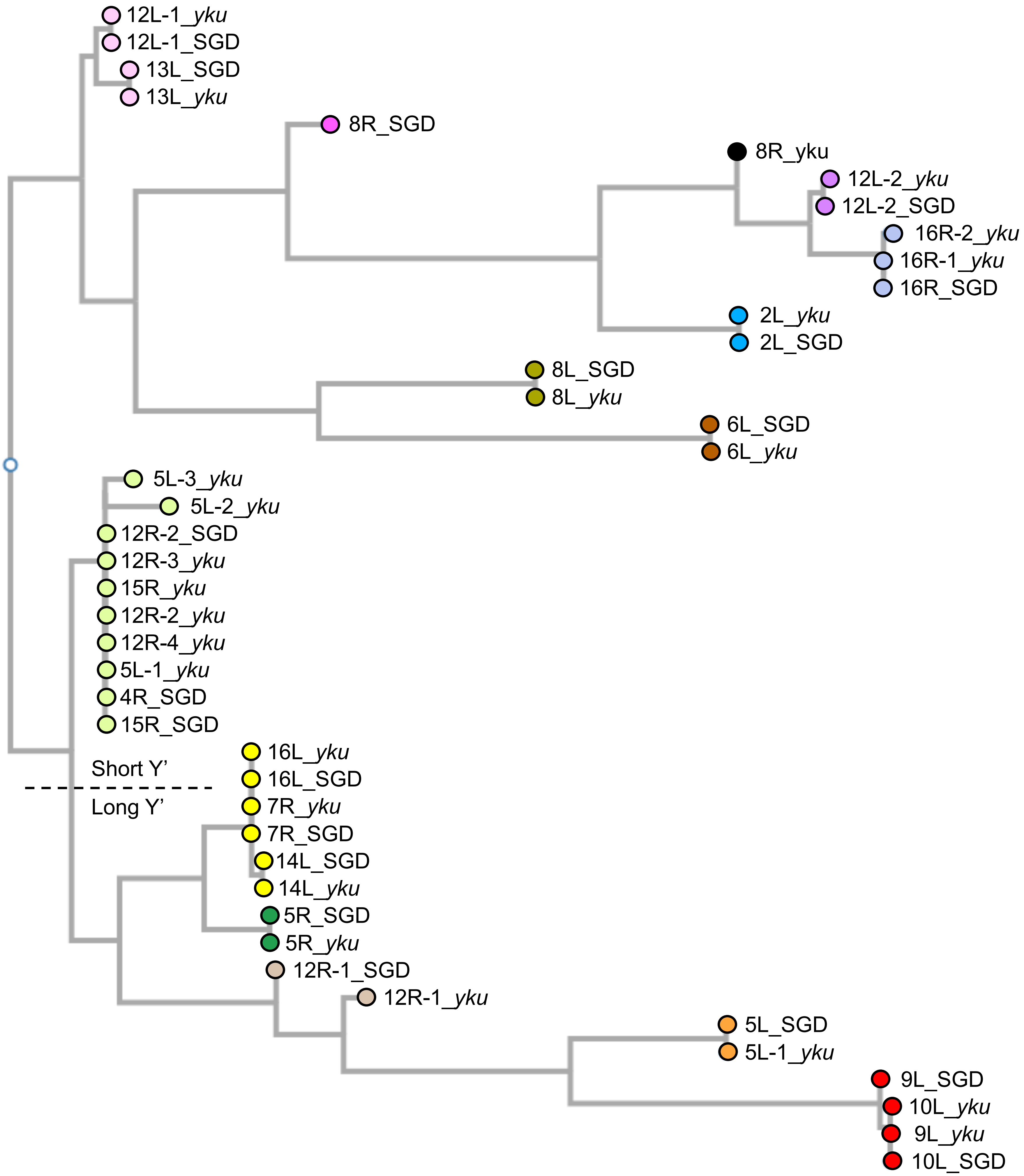

Supplement: Supplementary file 4 [file pgen.1012214.s004.pdf]
