# Supplementary material for: Telomeric amplicons of SUL1 and Y’ in yeast are generated by microhomology-mediated break induced replication occurring in cis
Source: PLoS Genet. 2026 Aug 20;22(8):e1012214. doi: 10.1371/journal.pgen.1012214 (PMC13515017; doi:10.1371/journal.pgen.1012214)

S5 Fig

A Y's in *yku70Δ*-A Read 1 (37672 bp) aligned with Y's of *yku70Δ* parent strain

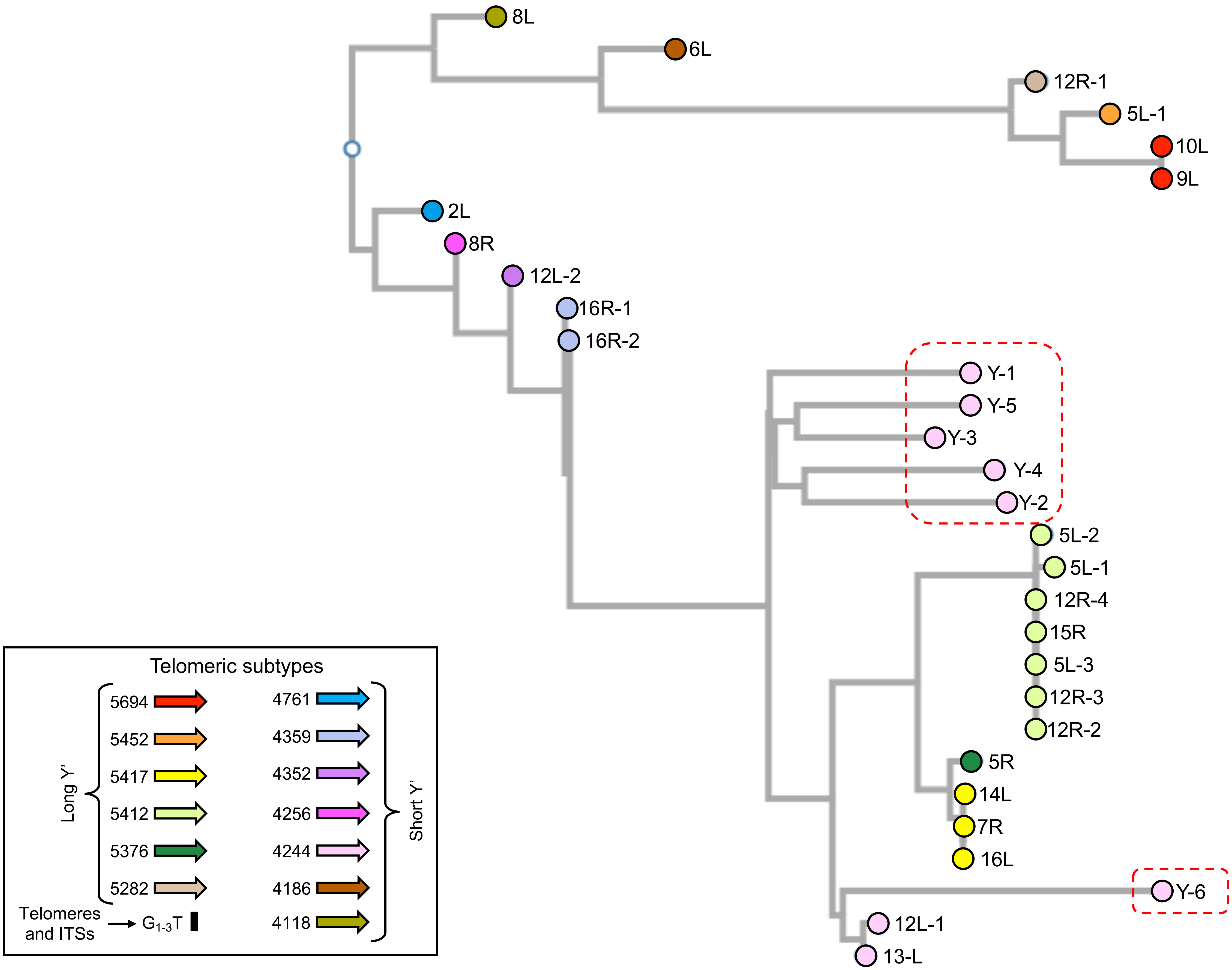

S5 Fig

B Y's in *yku70Δ*-A Read 2 (111251 bp) aligned with Y's in *yku70Δ* parent strain

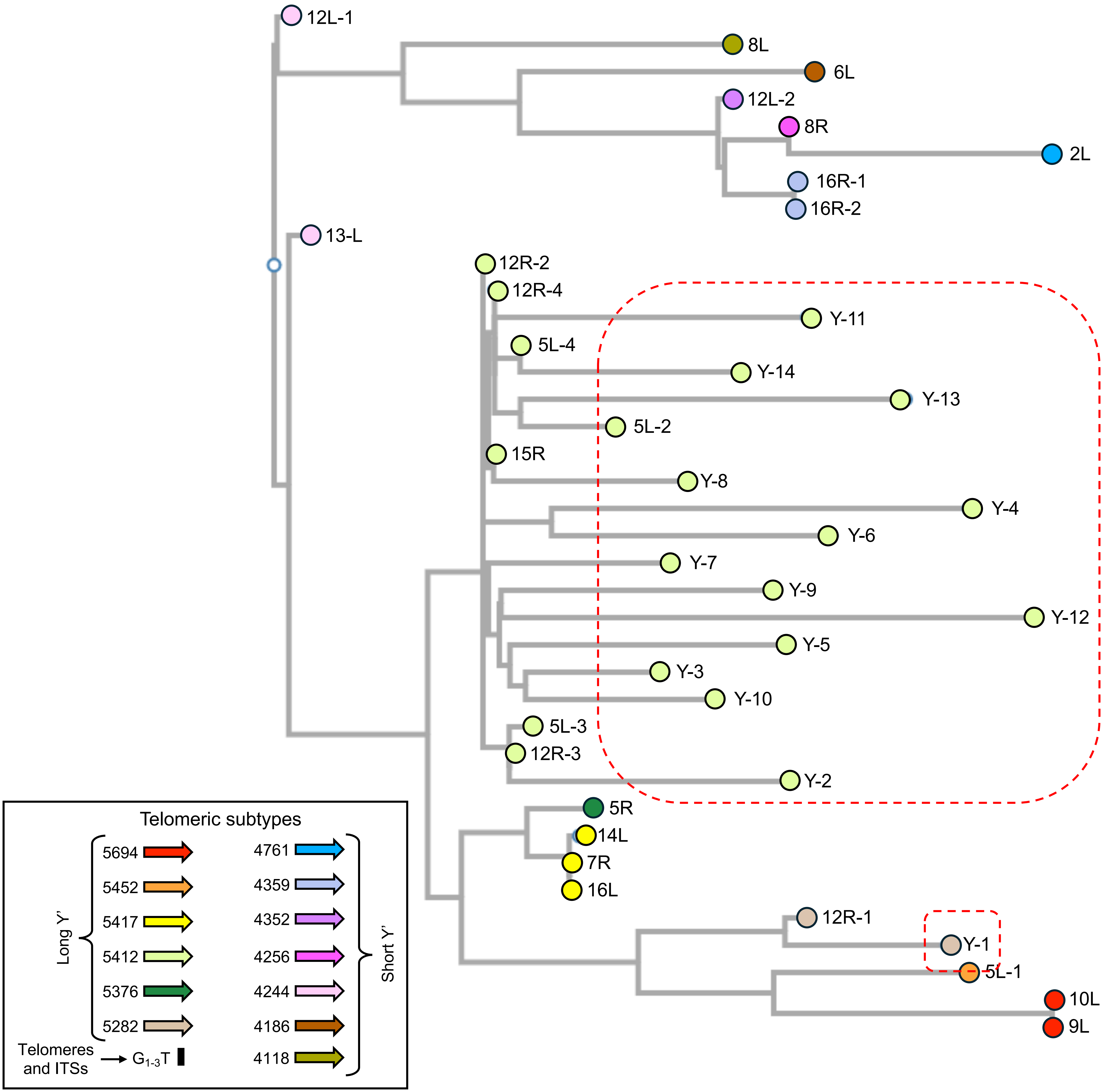

S5 Fig

C Y's in *yku70Δ*-A Read 3 (50690 bp) aligned with Y's in *yku70Δ* parent strain

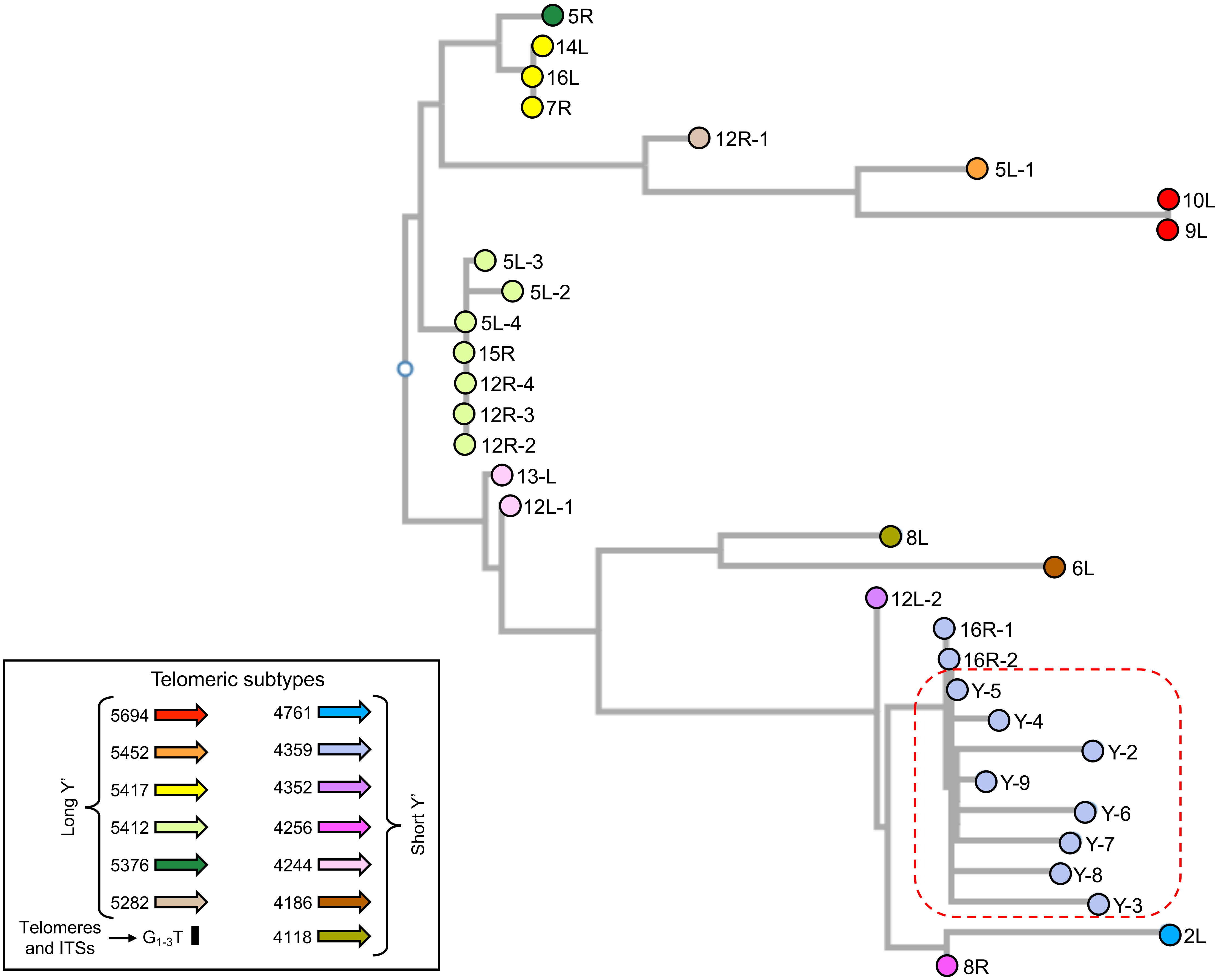

S5 Fig

D Y's in *yku70Δ*-A Read 4 (59060 bp) aligned with Y's in *yku70Δ* parent strain

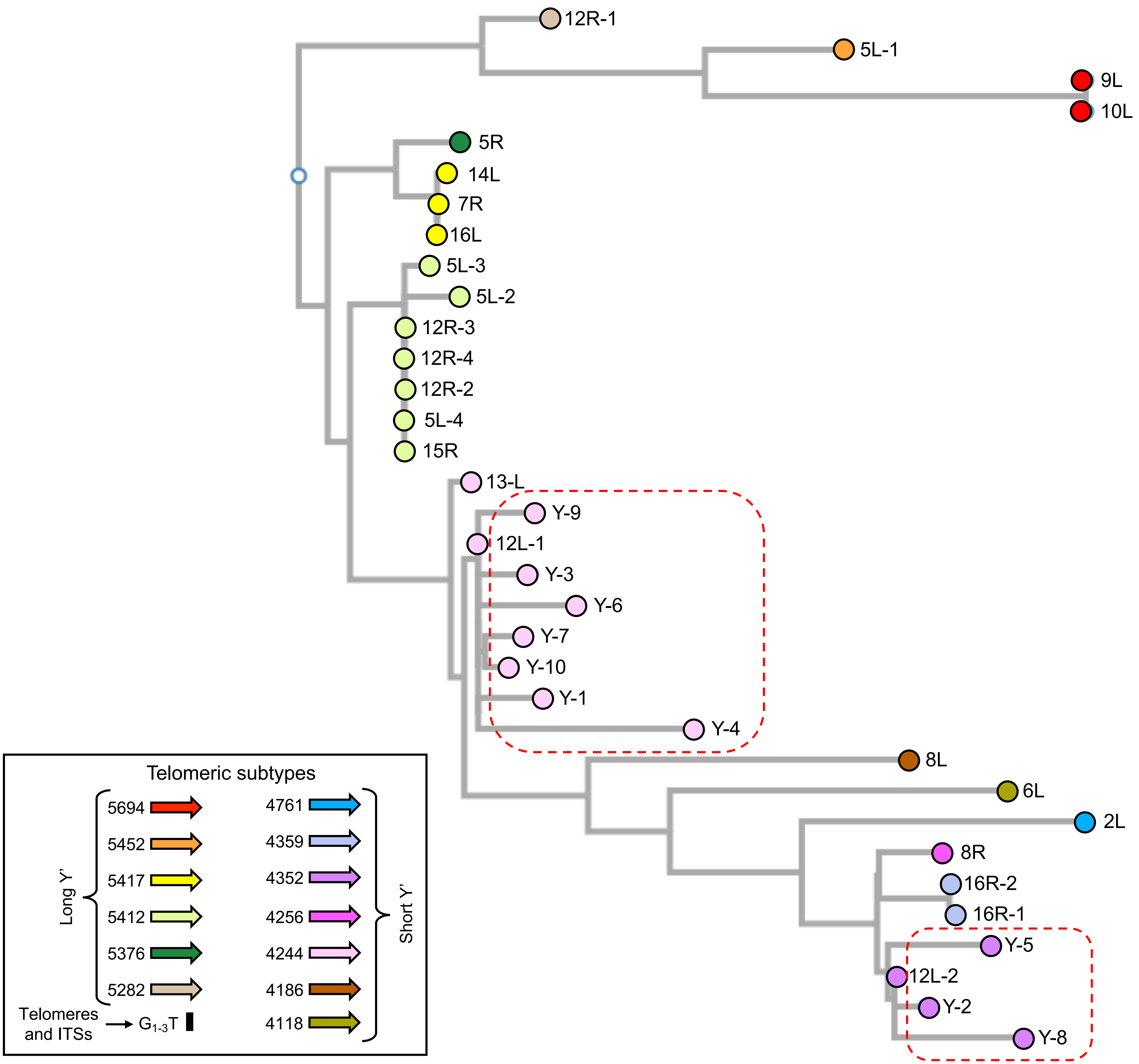

Supplement: Supplementary file 5 [file pgen.1012214.s005.pdf]
