# Supplementary material for: Telomeric amplicons of SUL1 and Y’ in yeast are generated by microhomology-mediated break induced replication occurring in cis
Source: PLoS Genet. 2026 Aug 20;22(8):e1012214. doi: 10.1371/journal.pgen.1012214 (PMC13515017; doi:10.1371/journal.pgen.1012214)
Supplement: Supplementary file 6 [file pgen.1012214.s006.pdf]

S6 Fig

A Read 1 (37672 bp) from *yku70Δ*–A

```
ITS-4      GTGTGGGTGTGGGTGTGGGTGTGTGGGTGTGGTGTGTGTGTG-------GGGTGTGG-TG
ITS-6      GTGTGGGTGTGGGTGTGGGTGTGTGGGTGTGGTGTGTGTGTGT------GGGTGTGGGTG
ITS-5      GTGTGGGTGTGGGTGTGGGTGTGTGGGTGTGGTGTGTGTGTG--------GGTGTGGGTG
ITS-7      ------GTGTGGGTGTGGGTGTGGGGGGGGGGTGTGTG------------GGTGTGGGTG
ITS-3      GTGTGGGTGTGGGTGTGGGTGTGTGGGTGTGGTGTGTGTGTGGGTGTGGGTGTGTGGGTG
                 ***************** *** * ********             ****** **

ITS-4      TG-TGTGTGGGTGTGGTGTGTGTGGGTGTGGGTGTGGGTGTGGTGTGTGGGTGTGGTGTG
ITS-6      TG-TGTGTGGGTGTGGTGTGTGTGGGTGTGGGTGTGGGTGTGGTGTGTGGGTGTGGTGTG
ITS-5      TG-TGTGTGGGTGTGGTGTGTGTGGGTGTGGGTGTGGGTGTGGTGTGTGGGTGTGGTGTG
ITS-7      TG-TGTGTGGGTGTGGTGTGTGTGGGTGTGGGTGTGGGTGTGGTGTGTGGGTGTGGTGTG
ITS-3      TGGTGTGTGGGTGTGGTGTGTGGGTGTGTGGGTGTGGGTGTGGTGTGTGGGTGTGGTGTG
           ** ******************* * ***********************************

ITS-4      TGGGTGTGGGTGTGTGGGTGTG-GTGTGGGTGTGGTGTGTGGGTGTGGTGTG--GTGTGT
ITS-6      TGGGTGTGGGTGTGTGGGTGTG-GTGTGGGTGTGGTGTGTGGGTGTGGTGTG--GTGTGT
ITS-5      TGGGTGTGGGTGTGTGGGTGTG-GTGTGGGTGTGGTGTGTGGGTGTGGTGTGTGGTGTGT
ITS-7      TGGGTGTGGGTGTGTGGGTGTG-GTGTGGGTGTGGTGTGTGGGTGTGGTGTG--GTGTGT
ITS-3      TGGGTGTGGGTGTG-GTGTGTGTGTGTGGGTGTGGTGTGTGTG---GGTGTG--GTGTGT
           ************** * ***** ****************** *   ******  ******

ITS-4      GGGTGTGGGTGTGGTGTGTGGG
ITS-6      GGGTGTGGGTGTGGTGTGTGGG
ITS-5      GGGTGTGGGTGTGGTGTGTGGG
ITS-7      GGGTGTGGGTGTGGTGTGTGGG
ITS-3      GGGTGTGGGTGTGGTGTGTGGG
           **********************
```

D Phylogenetic relationship of ITSs

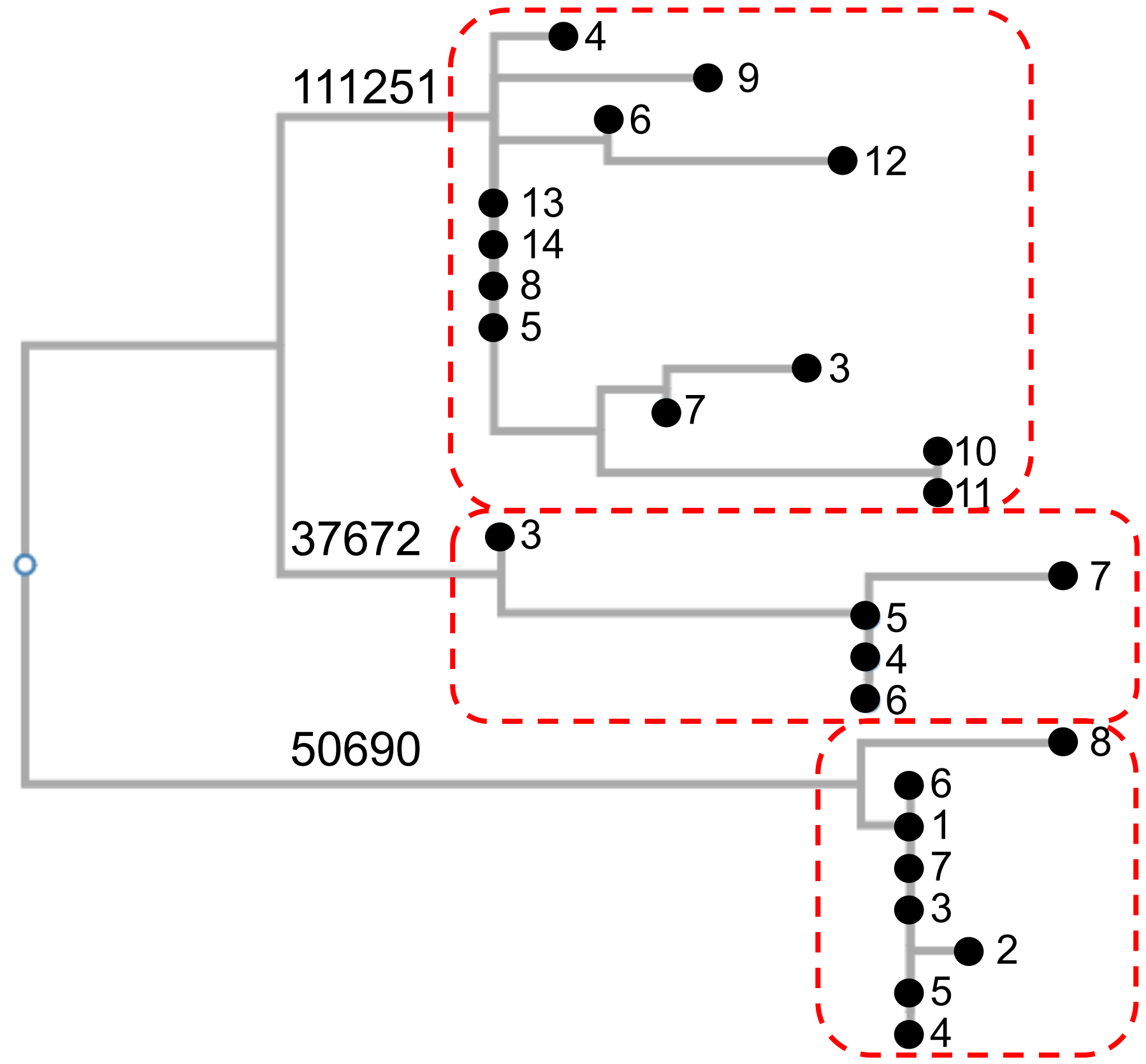

B Read 2 (111251 bp) from *yku70Δ*–A

```
ITS-6      GTGTGTGGGTGTGGTG---TGGGTGTGGTGTGTG-GGTGTGGGTGTGGGTGTGGG-T-TG
ITS-14     GTGTGTGGGTGTGGTG---TGGGTGTGGTGTGTG-GGTGTGGGTGTGGGTGTGGG-TGTG
ITS-4      GTGTGTGGGTGTGGTG---TGGGTGTGGTGTGTG-GGTGTGGGTGTGGGTGTGGGGTGTG
ITS-5      GTGTGTGGGTGTGGTG---TGGGTGTGGTGTGTG-GGTGTGGGTGTGGGTGTGGG-TGTG
ITS-8      GTGTGTGGGTGTGGTG---TGGGTGTGGTGTGTG-GGTGTGGGTGTGGGTGTGGG-TGTG
ITS-13     GTGTGTGGGTGTGGTG---TGGGTGTGGTGTGTG-GGTGTGGGTGTGGGTGTGGG-TGTG
ITS-9      -TGTGTGGGTGTGGTG---TGGGTGTGGTGTGTG-GGTGTGGGTGTGGGTGGTGGGTGTG
ITS-12     GTGTGTGGGTGTGGTG---TGGGTG--GTGTGTG-GGTGTGGGTGTGGGTGTGGGTGTGG
ITS-3      GTGTGTGGGTGTGGTG-TGTGGGTGT-GTGGGTGTGGTGTGTGTGTGGGTGTGGG-TGTG
ITS-7      GTGTGTGGGTGTGGTG-TGTGGGTGT-GTGGGTGTGGTGTGTGTGTGGGTGTGGG-TGTG
ITS-10     --GTGTGGGTGTGGTGGTGTGGGTGT-GTGTGGGTG-TG-GTGTGTGGGTGTGGG-TGTG
ITS-11     --GTGTGGGTGTGGTGGTGTGGGTGT-GTGT--GTG-TG-GTGTGTGGGTGTGGG-TGTG
             **************   ******  ***   * * ** * *********  **    *

ITS-6      GTGTGGTGTGGGTGTGGTGTGTGGGTGTGGGTGTGGGTGTGTGGGTGTGGTGTGTGGGTG
ITS-14     GTGTGGTGTGGGTGTGGTGTGTGGGTGTGGGTGTGGGTGTGTGGGTGTGGTGTGTGGGTG
ITS-4      GTGTGGTGTGGGTGTGGTGTGTGGGTGTGGGTGTGGGTGTGTGGGTGTGGTGTGTGGGTG
ITS-5      GTGTGGTGTGGGTGTGGTGTGTGGGTGTGGGTGTGGGTGTGTGGGTGTGGTGTGTGGGTG
ITS-8      GTGTGGTGTGGGTGTGGTGTGTGGGTGTGGGTGTGGGTGTGTGGGTGTGGTGTGTGGGTG
ITS-13     GTGTGGTGTGGGTGTGGTGTGTGGGTGTGGGTGTGGGTGTGTGGGTGTGGTGTGTGGGTG
ITS-9      GTGTGGTGTGGGTGTGGTGTGTGGGTGTGGGTGTGGGTGTGTGGGTGTGGTGTGTGGGTG
ITS-12     GTGTGGTGTGGGTGTGGTGTGTGGGTGTGTGTGTGGGTGTGTGGGTGTGGTGTGTGGGTG
ITS-3      GTGTGGTGTGGGTGTGGTGTGTGGGTGTGGGTGTGGGTGTGTGGGTGTGGTGTGTGGGTG
ITS-7      GTGTGGTGTGGGTGTGGTGTGTGGGTGTGGGTGTGGGTGTGTGGGTGTGGTGTGTGGGTG
ITS-10     GTGTGGTGTGGGTGTGGTGTGTGGGTGTGGGTGTGGGTGTGTGGGTGTGGTGTGTGGGTG
ITS-11     GTGTGGTGTGGGTGTGGTGTGTGGGTGTGGGTGTGGGTGTGTGGGTGTGGTGTGTGGGTG
           ***************************** ******************************

ITS-6      TGTGGGTGTGGTGTGTGTGTGGGTGTGGTGTGTGGGTGTGGTGTGTGT--
ITS-14     TGTGGGTGTGGTGTGTGTGTGGGTGTGGTGTGTGGGTGTGGTGTGTGT--
ITS-4      TGTGTGTGTGGTGTGTGTGTGGGTGTGGTGTGTGGGTGTGGTGTGTGT--
ITS-5      TGTGGGTGTGGTGTGTGTGTGGGTGTGGTGTGTGGGTGTGGTGTGTGT--
ITS-8      TGTGGGTGTGGTGTGTGTGTGGGTGTGGTGTGTGGGTGTGGTGTGTGT--
ITS-13     TGTGGGTGTGGTGTGTGTGTGGGTGTGGTGTGTGGGTGTGGTGTGTGT--
ITS-9      TGTGGGTGCGGTGTGTGTGTGGGTGTGGTGTGTGGGTGTGGTGTGTGT--
ITS-12     TGTGGGTGTGGTGTGTGTGTGGGTGTGGTGTGTGGGTGTGGTGTGTGTGT
ITS-3      TGTGGGTGTGGTGTGTGTGTGGGTGTGGTGTGTGGGTGGTGTGTGT----
ITS-7      TGTGGGTGTGGTGTGTGTGTGGGTGTGGTGTGTGGGTGTGGTGTGTGT--
ITS-10     TGTGGGTGTGGTGTGTGTGTGGGTGTGGTGTGTGGGTGTGGTGTGTGT--
ITS-11     TGTGGGTGTGGTGTGTGTGTGGGTGTGGTGTGTGGGTGTGGTGTGTGT--
           **** *** *****************************  ******
```

C Read 3 (50690 bp) from *yku70Δ*–A

```
ITS-1      GTGTGGGTGTGGTGGTGTGGG-TGTGTGTGGGTGTGGTGTGTGGGTGTGGTGTGTG---G
ITS-6      GTGTGGGTGTGGTGGTGTGGG-TGTGTGTGGGTGTGGTGTG--GGTGTGGTGTGTG---G
ITS-5      GTGTGGGTGTGGTGGTGTGGG-TGTGTGTGGGTGTGGTGTGTGGGTGTGGTGTGTGTGTG
ITS-7      GTGTGGGTGTGGTGGTGTGGG-TGTGTGTGGGTGTGGTGTGTGGGTGTGGTGTGTGTGTG
ITS-3      GTGTGGGTGTGGTGGTGTGGG-TGTGTGTGGGTGTGGTGTGTGGGTGTGGTGTGTGTGTG
ITS-4      GTGTGGGTGTGGTGGTGTGGGGTGTGTGTGGGTGTGGTGTGTGGGTGTGGTGTGTGTGTG
ITS-2      GTGTGGGTGTGGTGGTGTGGG-TGTGTGTGGGTG---TGTGTGTGTGTGGTGTGTGTGTG
ITS-8      GTGTGGGTGTGGTGGTGTGGG-TGTGTGTGGGTGTGGTGTGTGGGTGTGGTGTGTG----
           ********************* ************   ****   ************

ITS-1      GTGTGGGTGTGGTGTGGG-TGTGGTGTGGGTGTGGTGTGTGTGTGGGTGTGTGTGTGTGG
ITS-6      GTGTGGGTGTGGTGTGGGGTGTGGTGTGGGTGTGGTGTGTGTGTGGGTGTGTGTGTGTGG
ITS-5      GTGTGGGTGTGGTGTGGG--GTGGTGTGGGTGTGGTGTGTGTGTGGGTGTGTGTGTGTGG
ITS-7      GTGTGGGTGTGGTGTGGG-TGTGGTGTGGGTGT---GTGTGTGTGGGTGTGTGTGTGTGG
ITS-3      GTGTGGGTGTGGTGTGGG-TGTGGTGTGGGTGTGGTGTGTGTGTGGGTGTGTGTGTGTGG
ITS-4      GTGTGGGTGTGGTGTGGG-TGTGGTGTGGGTGTGGTGTGTGTGTGGGTGTGTGTGTGTGG
ITS-2      GTGTGGGTGTGGTGTGGG-TGTGGTGTGGGTGTGGTGTGTGTGTGGGTGTGTGTGTGTGG
ITS-8      -----GGTGTGGTGTGTG-TG-GGTGTGGGTGTGGTGTGGGTGTGG-TGTGTGTGTGTGG
                *********** *  * ***********   *** ****** *************

ITS-1      GTGTGGTGTGTGTGTGTGGGTGTGGTGTGTGTGGGTGTGGTGTGTGGGTGTGGGTG-TGG
ITS-6      GTGTGGTGTGTGTGTGTGGGTGTGGTGTGTGTGGGTGTGGTGTGTGGGTGTGGGTG-TGG
ITS-5      GTGTGGTGTGTGTGTGTGGGTGTGGTGTGTGTGGGTGTGGTGTGTGGGTGTGGGTGGTGG
ITS-7      GTGTGGTGTGTGTGTGTGGGTGTGGTGTGTGTGGGTGTGGTGTGTGGGTGTGGGTG-TGG
ITS-3      GTGTGGTGTGTGTGTGTGGGTGTGGTGTGTGTGGGTG--GTGTGTGGGTGTGGGTG-TGG
ITS-4      GTGTGGTGTGTGTGTGTGGGTGTGGTGTGTGTGGGTGTGGTGTGTGGGTGTGGGTG-TGG
ITS-2      GTGTGGTGTGTGTGTGTGGGTGTGGTGTGTGTGGGTGTGGTGTGTGGGTGTGGGTG-TGG
ITS-8      GTGTGGTGTGTGTGTGTGGGTGTGGTGTGTGTGGGTGTGGTGTGTGGGTGTGGGTG-TGG
           *************************************  ***************** ***

ITS-1      TGTGTGTGGGTGTGGTGTGTGGGT
ITS-6      TGTGTGTGGGTGTGGTGTGTGGGT
ITS-5      TGTGTGTGGGTGTGGTGTGTGGGT
ITS-7      TGTGTGTGGGTGTGGTGTGTGGGT
ITS-3      TGTGTGTGGGTGTGGTGTGTGGGT
ITS-4      TGTGTGTGGGTGTGGTGTGTGGGT
ITS-2      TGTGTGTGGGTGTGGTGTGTGGGT
ITS-8      TGTGTGTGGGTGTGGTGTGTGGGT
           ************************
```
