# Supplementary material for: Telomeric amplicons of SUL1 and Y’ in yeast are generated by microhomology-mediated break induced replication occurring in cis
Source: PLoS Genet. 2026 Aug 20;22(8):e1012214. doi: 10.1371/journal.pgen.1012214 (PMC13515017; doi:10.1371/journal.pgen.1012214)
Supplement: Supplementary file 7 [file pgen.1012214.s007.pdf]

S7 Fig

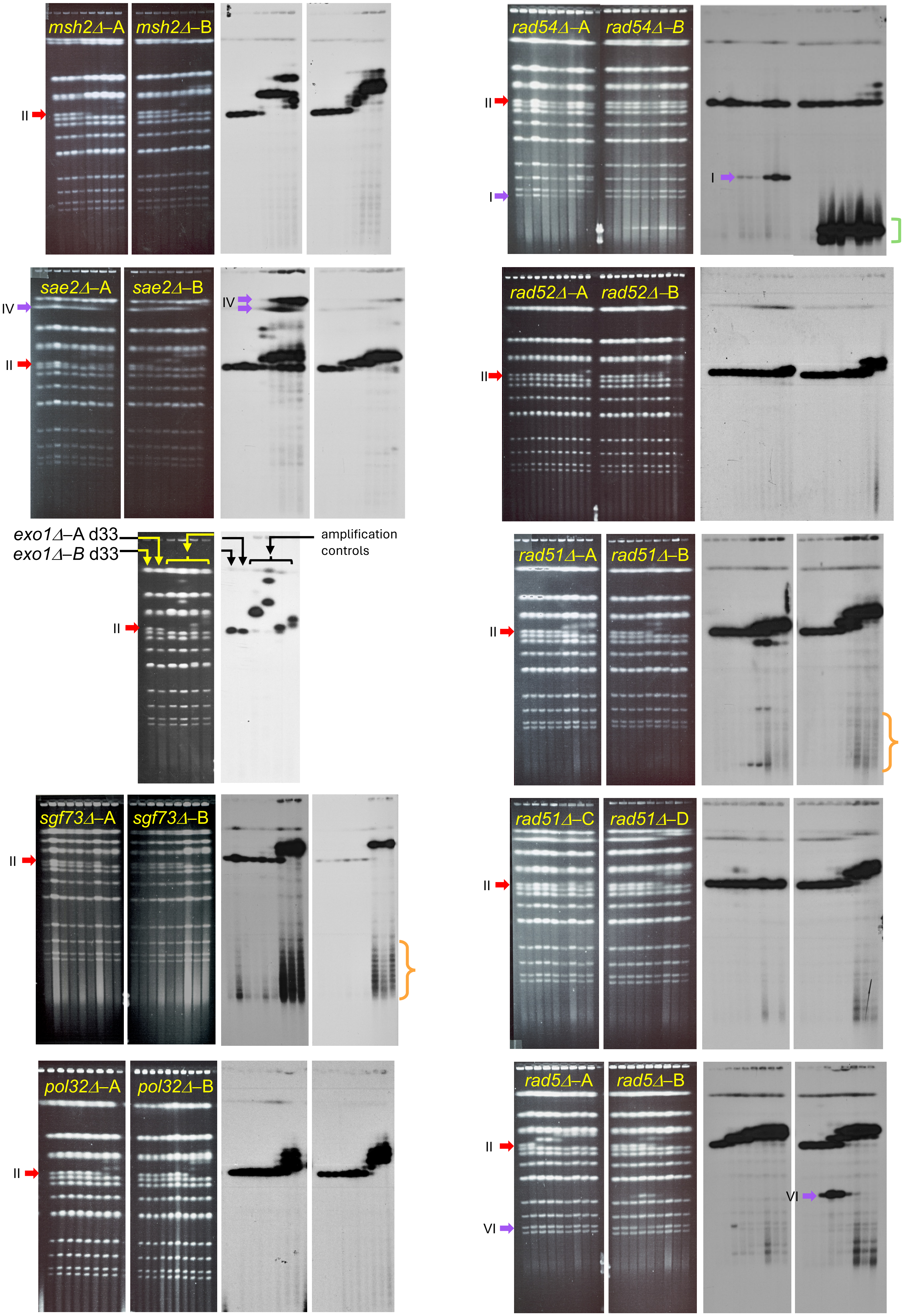

msh2Δ–A
msh2Δ–B
II
rad54Δ–A
rad54Δ–B
II
I
I
sae2Δ–A
sae2Δ–B
IV
II
IV
rad52Δ–A
rad52Δ–B
II
exo1Δ–A d33
exo1Δ–B d33
amplification controls
II
rad51Δ–A
rad51Δ–B
II
sgf73Δ–A
sgf73Δ–B
II
rad51Δ–C
rad51Δ–D
II
pol32Δ–A
pol32Δ–B
II
rad5Δ–A
rad5Δ–B
II
VI
VI
