# Supplementary material for: Telomeric amplicons of SUL1 and Y’ in yeast are generated by microhomology-mediated break induced replication occurring in cis
Source: PLoS Genet. 2026 Aug 20;22(8):e1012214. doi: 10.1371/journal.pgen.1012214 (PMC13515017; doi:10.1371/journal.pgen.1012214)
Supplement: Supplementary file 8 [file pgen.1012214.s008.pdf]

S8 Fig

A

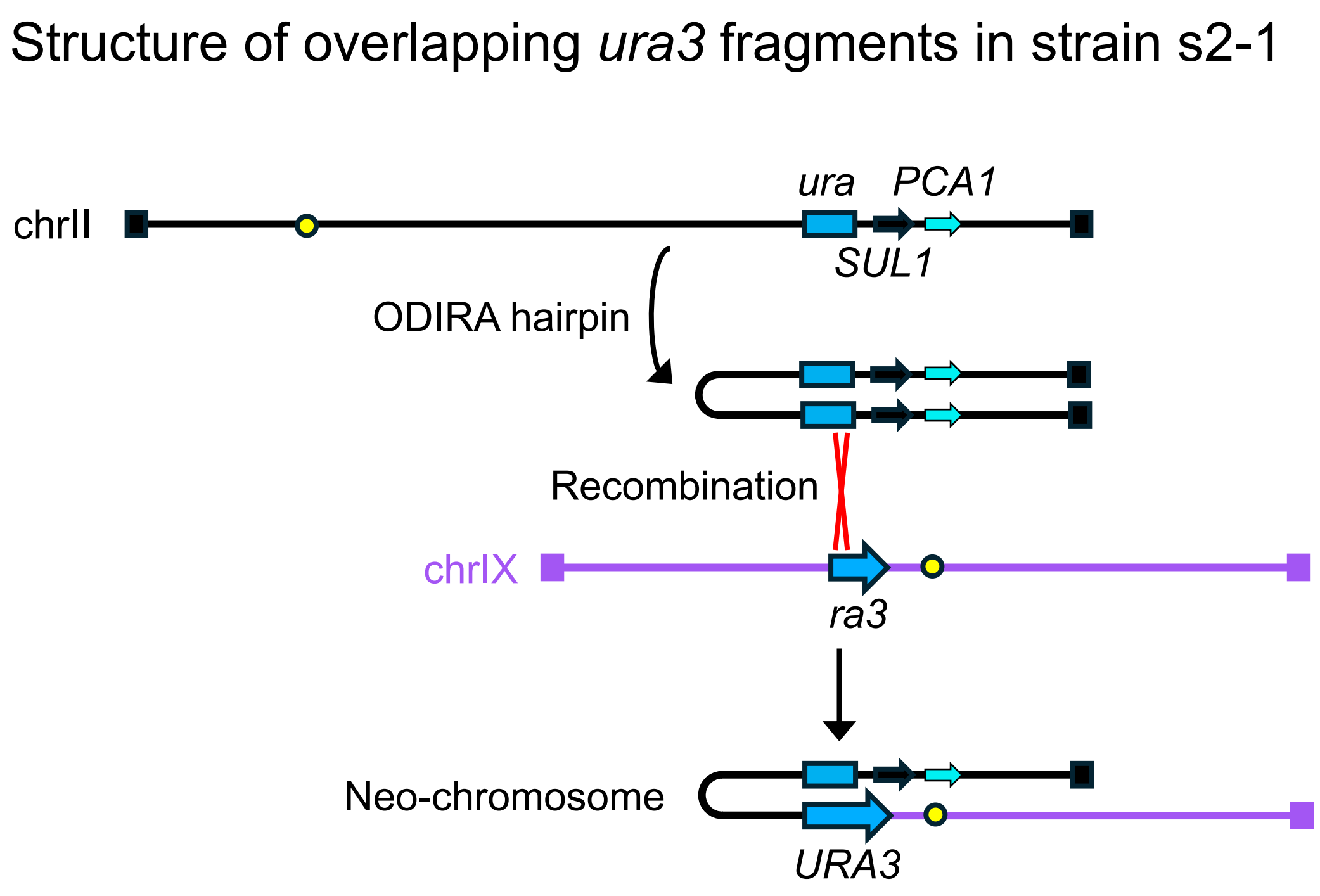
Structure of overlapping ura3 fragments in strain s2-1
chrII
ura
PCA1
SUL1
ODIRA hairpin
Recombination
chrIX
ra3
Neo-chromosome
URA3

B

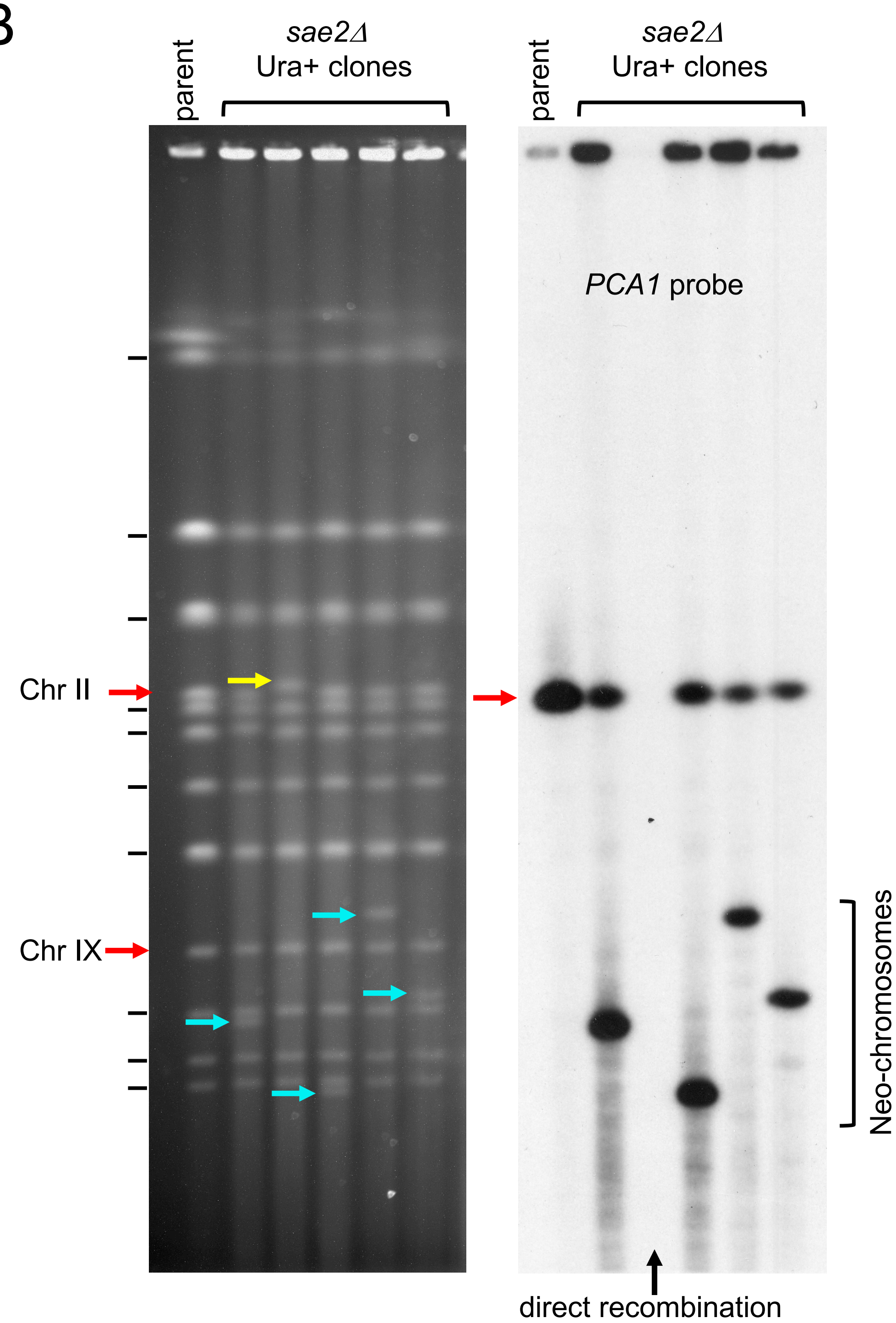
parent
sae2Δ
Ura+ clones
Chr II
Chr IX
PCA1 probe
Neo-chromosomes
direct recombination

C

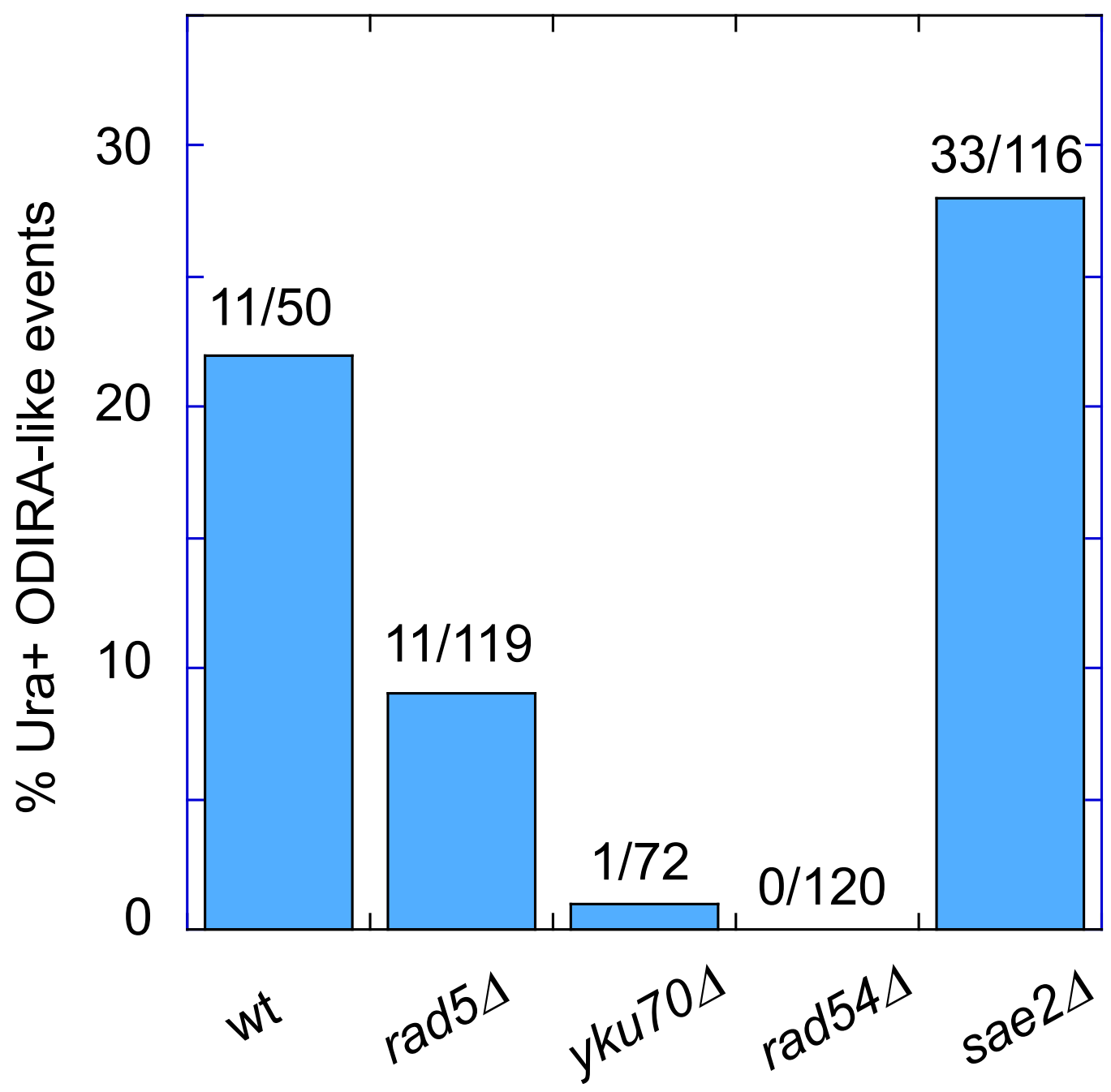
% Ura+ ODIRA-like events
0
10
20
30
11/50
11/119
1/72
0/120
33/116
wt
rad5Δ
yku70Δ
rad54Δ
sae2Δ
