# Supplementary material for: Telomeric amplicons of SUL1 and Y’ in yeast are generated by microhomology-mediated break induced replication occurring in cis
Source: PLoS Genet. 2026 Aug 20;22(8):e1012214. doi: 10.1371/journal.pgen.1012214 (PMC13515017; doi:10.1371/journal.pgen.1012214)
Supplement: Supplementary file 9 [file pgen.1012214.s009.pdf]

S9 Fig

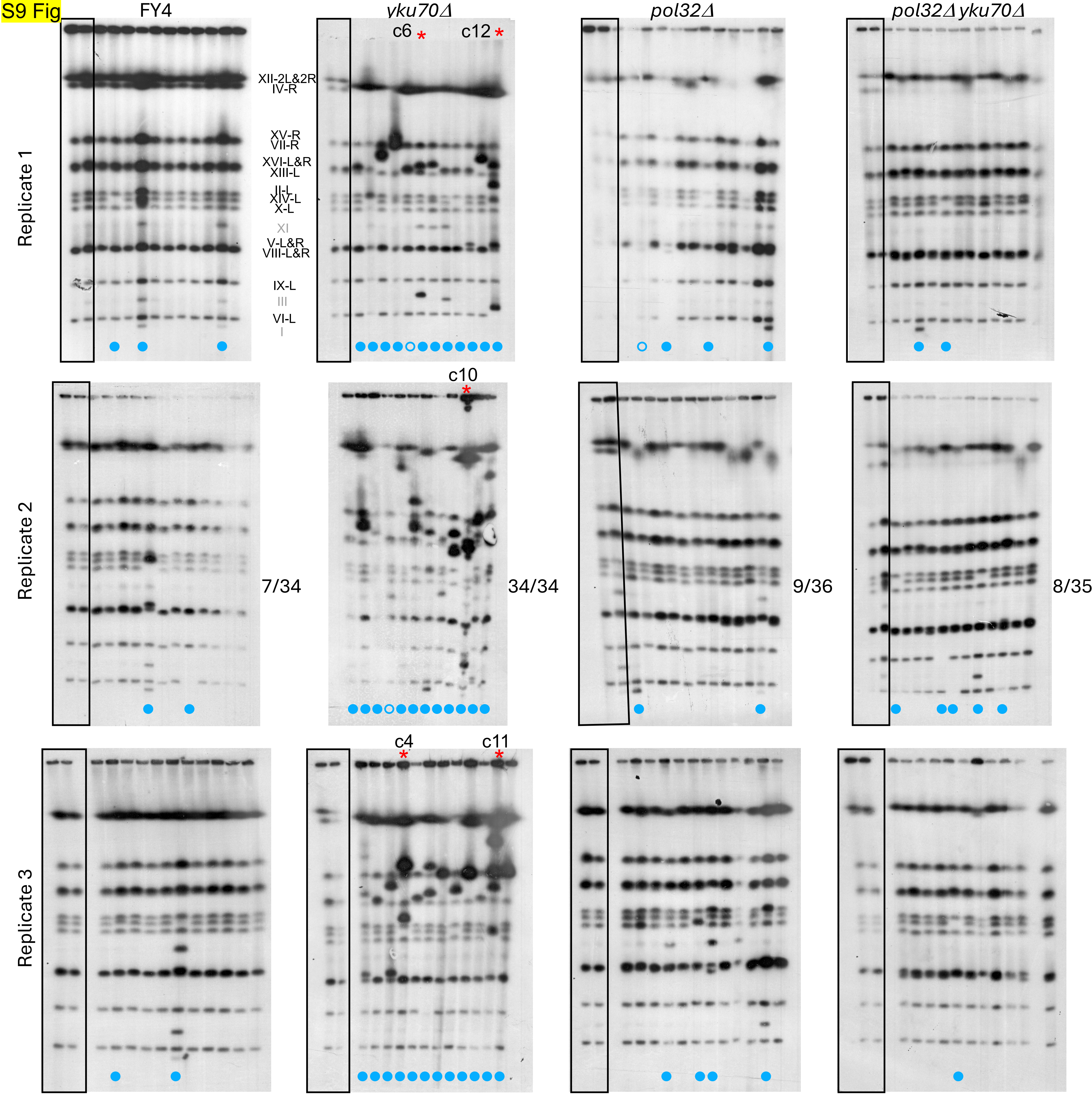

FY4
yku70Δ
pol32Δ
pol32Δ yku70Δ
c6 *
c12 *
XII-2L&2R
IV-R
XV-R
VII-R
XVI-L&R
XIII-L
II-L
XIV-L
X-L
XI
V-L&R
VIII-L&R
IX-L
III
VI-L
I
Replicate 1
c10 *
Replicate 2
7/34
34/34
9/36
8/35
c4 *
c11 *
Replicate 3
