# Supplementary material for: Telomeric amplicons of SUL1 and Y’ in yeast are generated by microhomology-mediated break induced replication occurring in cis
Source: PLoS Genet. 2026 Aug 20;22(8):e1012214. doi: 10.1371/journal.pgen.1012214 (PMC13515017; doi:10.1371/journal.pgen.1012214)

S10 Fig

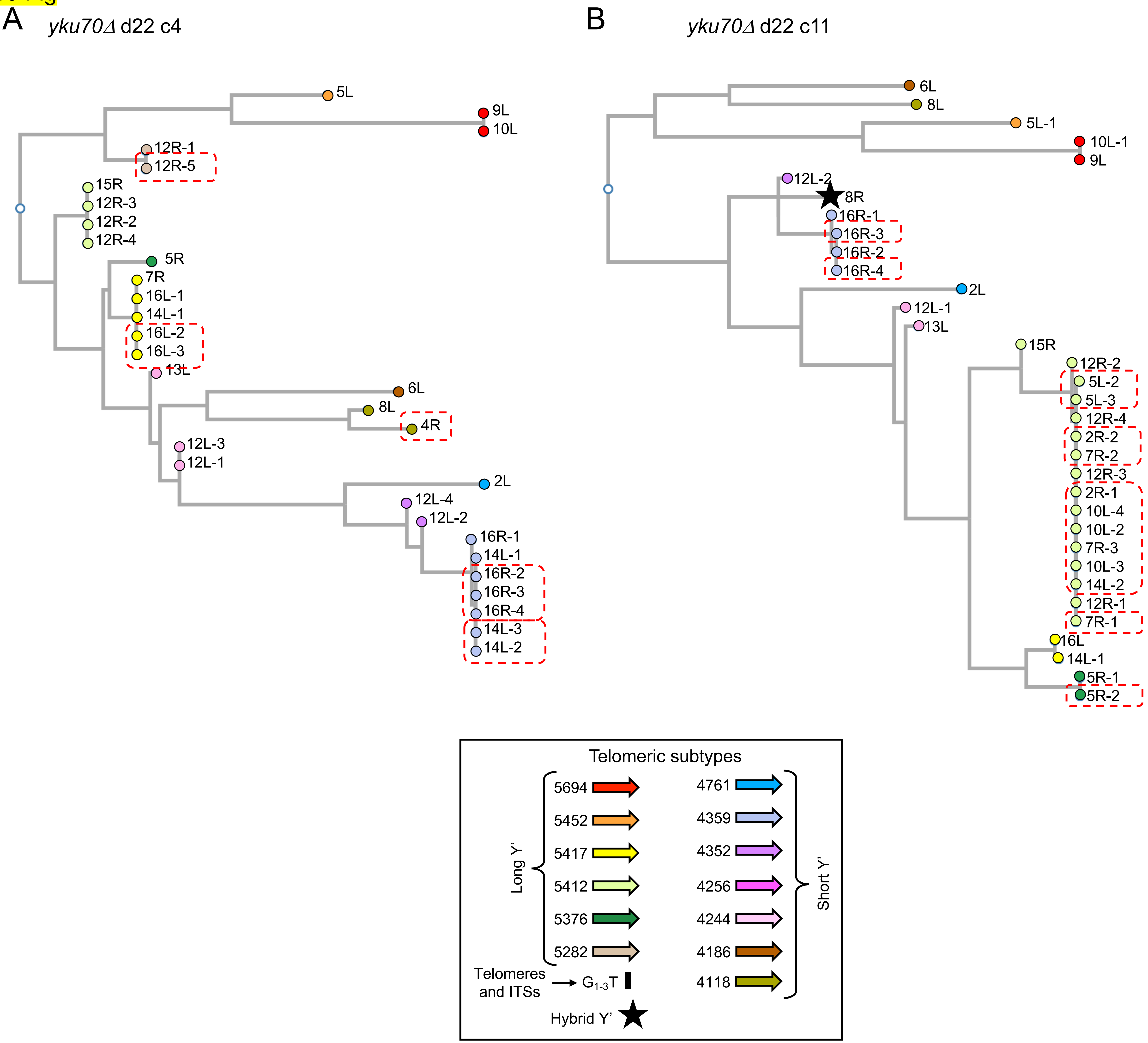
A
yku70Δ d22 c4
5L
9L
10L
12R-1
12R-5
15R
12R-3
12R-2
12R-4
5R
7R
16L-1
14L-1
16L-2
16L-3
13L
6L
8L
4R
12L-3
12L-1
2L
12L-4
12L-2
16R-1
14L-1
16R-2
16R-3
16R-4
14L-3
14L-2
B
yku70Δ d22 c11
6L
8L
5L-1
10L-1
9L
12L-2
8R
16R-1
16R-3
16R-2
16R-4
2L
12L-1
13L
15R
12R-2
5L-2
5L-3
12R-4
2R-2
7R-2
12R-3
2R-1
10L-4
10L-2
7R-3
10L-3
14L-2
12R-1
7R-1
16L
14L-1
5R-1
5R-2
Telomeric subtypes
Long Y'
5694
5452
5417
5412
5376
5282
Short Y'
4761
4359
4352
4256
4244
4186
4118
Telomeres and ITSs
$G_{1-3}T$
Hybrid Y'

S10 Fig

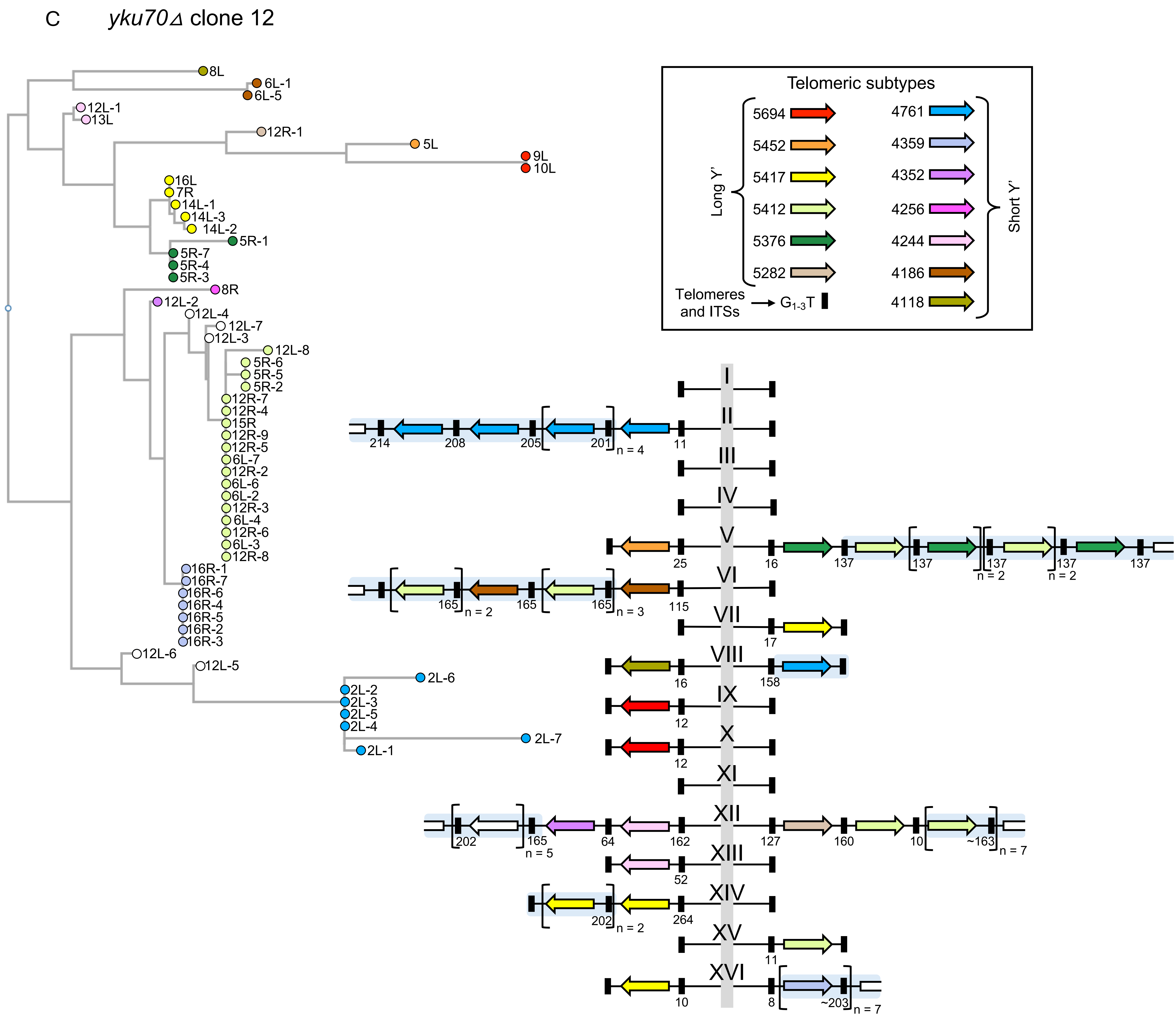
C
yku70Δ clone 12
8L
6L-1
6L-5
12L-1
13L
12R-1
5L
9L
10L
16L
7R
14L-1
14L-3
14L-2
5R-1
5R-7
5R-4
5R-3
8R
12L-2
12L-4
12L-7
12L-3
12L-8
5R-6
5R-5
5R-2
12R-7
12R-4
15R
12R-9
12R-5
6L-7
12R-2
6L-6
6L-2
12R-3
6L-4
12R-6
6L-3
12R-8
16R-1
16R-7
16R-6
16R-4
16R-5
16R-2
16R-3
12L-6
12L-5
2L-6
2L-2
2L-3
2L-5
2L-4
2L-7
2L-1
Telomeric subtypes
Long Y'
5694
5452
5417
5412
5376
5282
4761
4359
4352
4256
4244
4186
4118
Short Y'
Telomeres and ITSs
G1-3T
I
II
III
IV
V
VI
VII
VIII
IX
X
XI
XII
XIII
XIV
XV
XVI
214
208
205
201
n = 4
11
25
16
137
137
137
137
137
n = 2
n = 2
165
n = 2
165
165
n = 3
115
17
16
158
12
12
202
165
n = 5
64
162
127
160
10
~163
n = 7
52
202
n = 2
264
11
10
8
~203
n = 7

S10 Fig

D *yku70Δ* clone 10

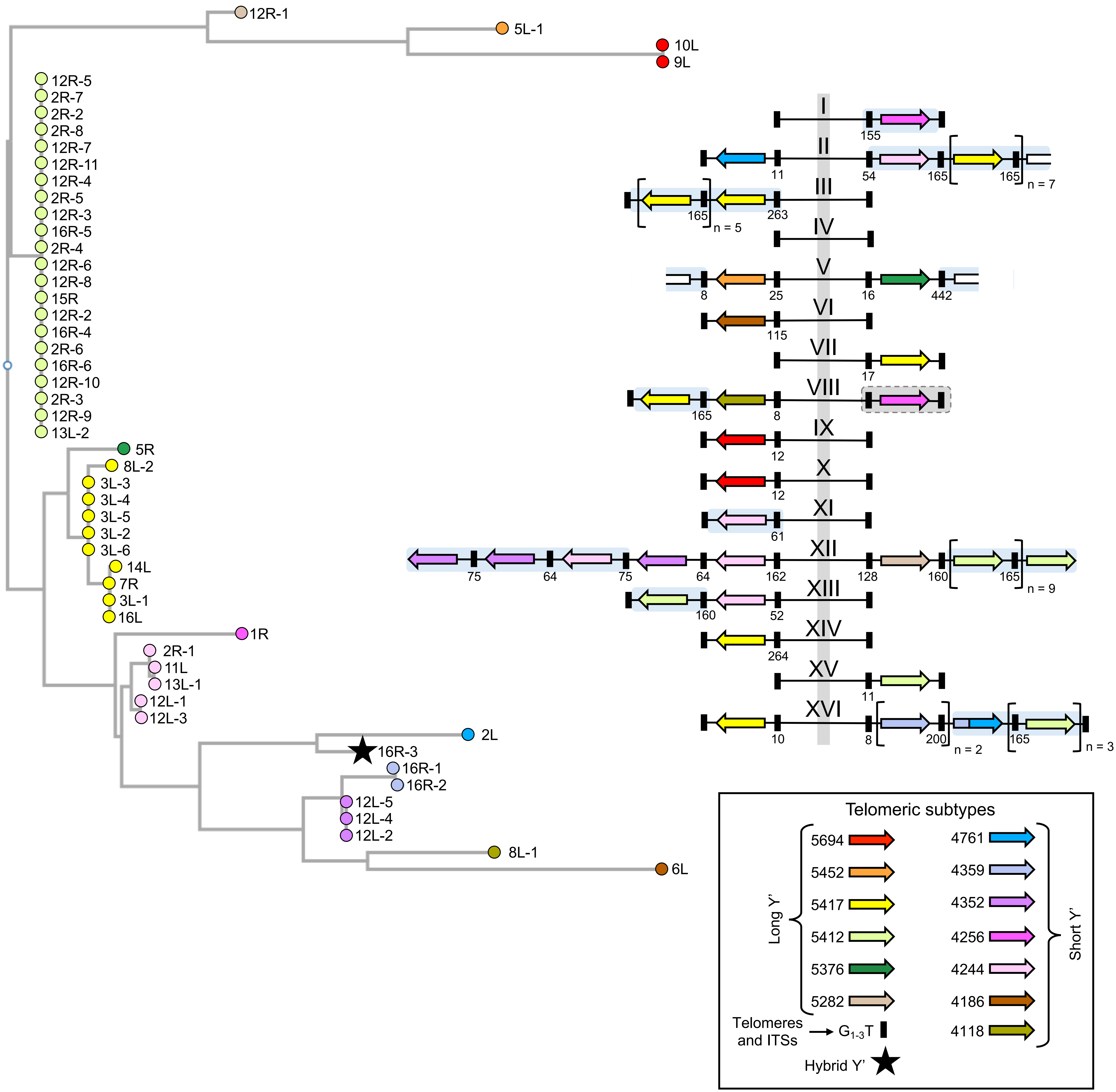

S10 Fig

E *yku70Δ* clone 6

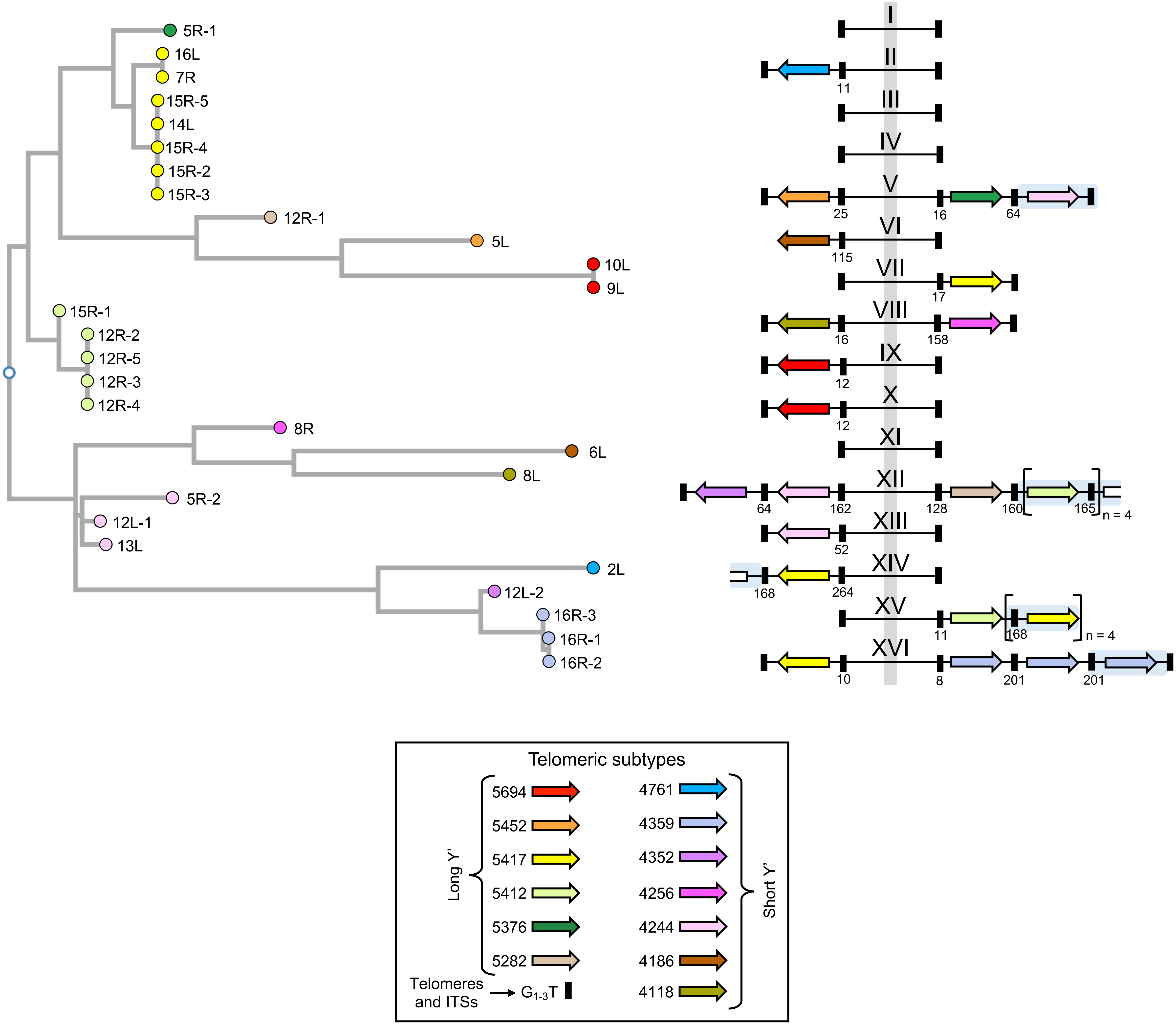

Supplement: Supplementary file 10 [file pgen.1012214.s010.pdf]
