# Supplementary material for: Telomeric amplicons of SUL1 and Y’ in yeast are generated by microhomology-mediated break induced replication occurring in cis
Source: PLoS Genet. 2026 Aug 20;22(8):e1012214. doi: 10.1371/journal.pgen.1012214 (PMC13515017; doi:10.1371/journal.pgen.1012214)
Supplement: Supplementary file 11 [file pgen.1012214.s011.pdf]

S11 Fig

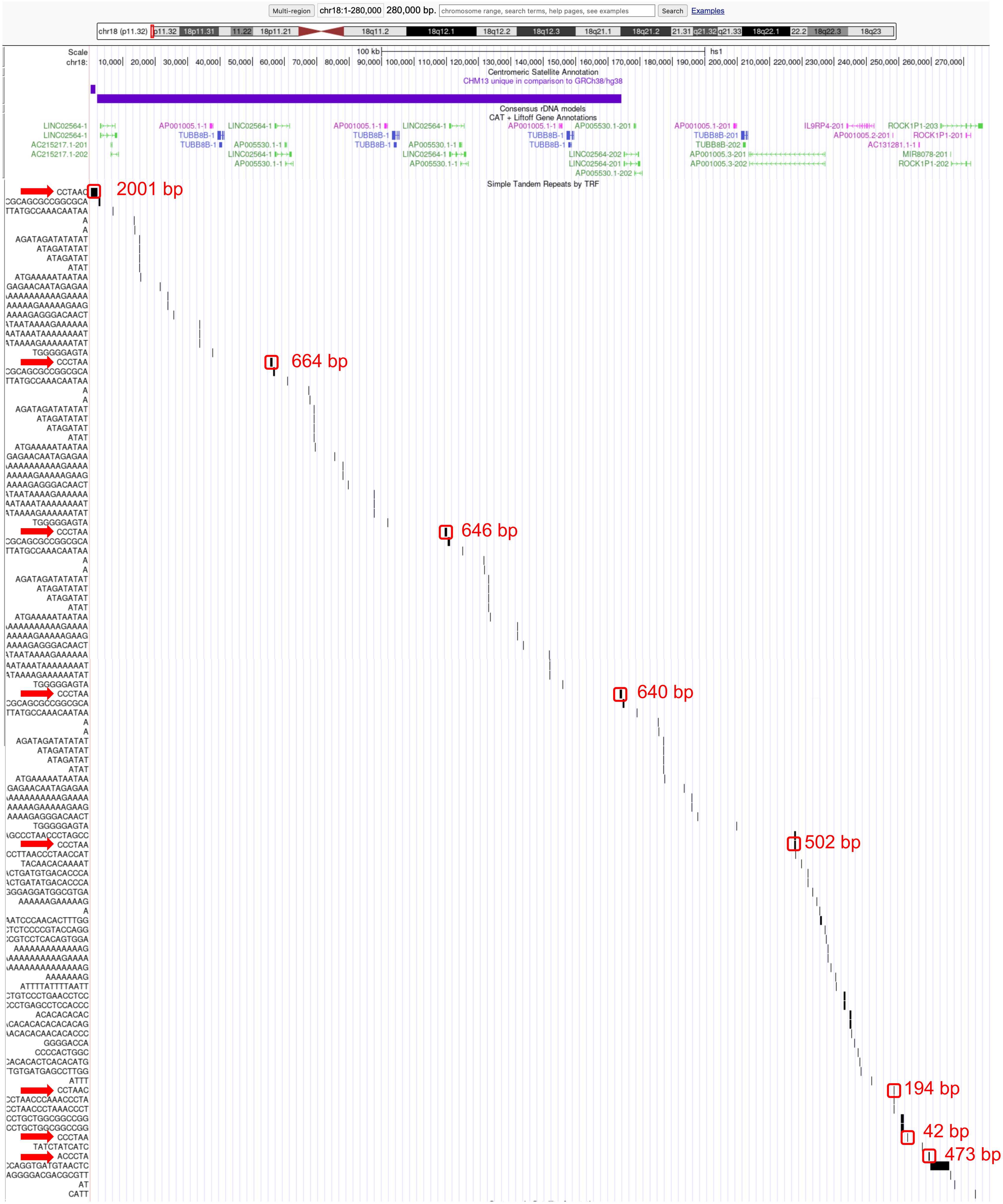

Multi-region
chr18:1-280,000
280,000 bp.
chromosome range, search terms, help pages, see examples
Search
Examples
chr18 (p11.32)
Scale
chr18:
100 kb
hs1
Centromeric Satellite Annotation
CHM13 unique in comparison to GRCh38/hg38
Consensus rDNA models
CAT + Liftoff Gene Annotations
LINC02564-1
AC215217.1-201
AC215217.1-202
AP001005.1-1
TUBB8B-1
AP005530.1-1
AP005530.1-201
LINC02564-202
LINC02564-201
AP005530.1-202
AP001005.1-201
TUBB8B-201
TUBB8B-202
AP001005.3-201
AP001005.3-202
IL9RP4-201
AP001005.2-201
AC131281.1-1
ROCK1P1-203
ROCK1P1-201
MIR8078-201
ROCK1P1-202
Simple Tandem Repeats by TRF
2001 bp
664 bp
646 bp
640 bp
502 bp
194 bp
42 bp
473 bp
CCTAAC
CCCTAA
ACCCTA
TGGGGGAGTA
AGATAGATATATAT
ATAGATATAT
ATAGATAT
ATAT
ATGAAAAATAATAA
GAGAACAATAGAGAA
AAAAGAGGGACAACT
TACAACACAAAAT
AAAAAAGAAAAAG
AAAAAAAG
ATTTTATTTTAATT
ACACACACAC
GGGGACCA
CCCCACTGGC
ATTT
TATCTATCATC
AT
CATT
